# Orthodontic and dentofacial orthopedic treatments in patients with ectodermal dysplasia: a systematic review

**DOI:** 10.1186/s13023-022-02533-0

**Published:** 2022-10-17

**Authors:** Marina Cerezo-Cayuelas, Amparo Pérez-Silva, Clara Serna-Muñoz, Ascensión Vicente, Yolanda Martínez-Beneyto, Inmaculada Cabello-Malagón, Antonio José Ortiz-Ruiz

**Affiliations:** 1 Department of Integrated Pediatric Dentistry, School of Dentistry, Biomedical Research Institute of Murcia, Morales Meseguer Hospital, University of Murcia, Avda. Marqués de los Vélez S/N, 30008 Murcia, Spain; 2 Orthodontics Unit, University Dental Clinic, Biomedical Research Institute of Murcia, University of Murcia, 30008 Murcia, Spain; 3 Department of Preventive and Community Dentistry, School of Dentistry, Biomedical Research Institute of Murcia, University of Murcia, 30008 Murcia, Spain

**Keywords:** Ectodermal dysplasia, Orthodontic treatment, Dentofacial orthopedic treatment, Anhidrotic ectodermal dysplasia, Hidrotic ectodermal dysplasia, Hypohidrotic ectodermal dysplasia, Anodontia, Hypodontia, Maxillary atrophy

## Abstract

**Objective:**

The objective of this systematic review was to determine the orthodontic and dentofacial orthopedic treatments carried out in patients with ectodermal dysplasia to facilitate functional and aesthetic rehabilitation.

**Methods:**

The systematic review was conducted in accordance with the Preferred Reporting Items for Systematic Reviews and Meta-analysis statement. We systematically searched PubMed, Web of Science, Scopus, Scielo, LILACS, EBSCOhost and Embase databases up to 6 January 2022. We included articles describing patients with any type of ectodermal dysplasia who received orthodontic or dentofacial orthopedic treatment to facilitate functional and aesthetic oral rehabilitation. The search was not restricted by language or year of publication. The quality of the studies was assessed using the Joanna Briggs Institute Quality Assessment Scale of the University of Adelaide for case series and case reports. The review was registered at the University of York Centre for reviews (CRD42021288030).

**Results:**

Of the initial 403 studies found, 29 met the inclusion criteria. After applying the quality scale, 23 were left for review—21 case reports and 2 case series. The initial age of patients ranged from 34 months to 24 years. Thirteen studies were on hypohidrotic and/or anhidrotic ectodermal dysplasia, of which two were X-chromosome linked. In one study, the patient had Wiktop syndrome, and in nine the type of ectodermal dysplasia was not specified. The duration of treatment was 7 weeks to 10 years. The treatments described were: fixed orthodontic appliances or simple acrylic plates designed for tooth movement, including leveling and aligning, closing of diastemata, retraction of impacted teeth in the dental arch; clear aligners; fixed and/or removable appliances for the correction of skeletal and/or dentoalveolar relationships; palatal expanders in combination with face masks for orthopedic traction of the maxilla; and orthognathic surgery. Only three studies provided cephalometric data.

**Conclusion:**

The level of evidence of the articles reviewed was low and most orthopedic and dentofacial orthodontic treatments described were focused on correcting dental malpositioning and jaw asymmetries and not on stimulating growth from an early age. Studies with greater scientific evidence are needed to determine the best treatment for these patients.

**Supplementary Information:**

The online version contains supplementary material available at 10.1186/s13023-022-02533-0.

## Introduction

Ectodermal dysplasia (ED; ORPHA:79373) comprises a large, diverse group of over 200 disorders. ED was defined by the Ectodermal Dysplasias Classification Working Group as a group of genetic conditions that affect the development and/or homeostasis of two or more ectodermal derivatives, including hair, teeth, nails, and some glands; their genetic causes and clinical phenotypes are heterogeneous [1]. The group proposed a new classification, that aims to be more useful than the classical clinical one [2, 3], based on molecular pathways, and integrating clinical and molecular information (gene, molecular pathway, and/or protein function). The categories are the EDA/NFKappaB pathway, the WNT pathway, the TP63 pathway, the structure group and other/unknown [1].

The overall prevalence of ED syndromes varies [4], although it may be close to 6–9/10,000 [5]. Hypohidrotic ectodermal dysplasia (HED; ORPHA:238468; ICD-10: Q82.4; OMIM: 129490, 224900, 300291, 305100, 612132, 614940, 614941) is the most common form of ED and is estimated to affect at least 1 in 5,000–10,000 newborns, with a prevalence of 1–9/100,000 [6].

HED is caused by mutations in the genes of the EDA/NFKappaB pathway (EDA, EDAR, EDARADD, WNT10A), which are necessary for the correct development of various ectodermal structures, in 90% of cases [7]. It affects the skin, hair, nails, sweat glands and teeth. Patients with HED have a characteristic physical appearance: the scalp and body hair are sparse and light in color (hypotrichosis), and patients have soft, hypopigmented skin, sunken cheeks, saddle nose, a frontal protrusion, prominent supraorbital crests, periorbital hyperpigmentation, low set ears, dysplastic nails, everted lips, prominent chin and reduced lower facial height with the lower third of the face reduced, giving these patients an aged appearance. Hyperkeratosis may affect the palms of the hands and soles of the feet, and women often have aplastic or hypoplastic mammary glands [8].

The most common form of HED, previously referred to as Christ-Siemens-Touraine syndrome, have mutations or deletions in the ectodysplasin gene and it is inherited as an X-linked condition (XHED; ORPHA:181; ICD-10: Q82.4; OMIM: 305100). XHED, with a prevalence of 1–9/1 000 000 [5], presents a clinical picture of hypotrichosis, hypohidrosis, oligodontia, and a predisposition to respiratory disorders [9].

Other types of ED include anhidrotic ectodermal dysplasia, in which the sweat and sebaceous glands are absent, and hidrotic ectodermal dysplasia or Clouston syndrome (ORPHA:189; ICD-10: Q82.8; OMIM: 129500), which is autosomal dominant, where these glands develop normally [8]. ED patients present a wide spectrum of orofacial dysfunctions: alterations in the teeth (almost 100%), chewing and swallowing (82.6%), dryness of the mouth (45.7%), speech (43.5%), hoarse voice, etc. [10, 11]**.**

The teeth are one of the four ectodermal structures included in the clinical classification proposed by Pinheiro and Freire-Maia (hair, teeth, nails, and sweat glands [3]. In fact, many children with ED are diagnosed after a first dental examination when there is a significant delay in the eruption of the primary teeth or when the first tooth appears in an atypical shape [12].

Agenesis is very common in all forms of ED, and different patterns of dental agenesis are observed (without considering the third molar): hypodontia (if < 6 teeth are missing), oligodontia (if ≥ 6 teeth are missing) and anodontia (total absence of permanent teeth) [13]. The most frequent missing teeth are the maxillary lateral incisors and first premolars and the mandibular incisors and first premolars. The permanent teeth most often present are the maxillary central incisors, first maxillary molars, first mandibular molars, and canines in both jaws. The anterior teeth are usually conical or tapered and the primary molars are sometimes ankylosed due to the absence of permanent premolars. Other dental alterations presented by patients with ED are taurodontism, enamel hypoplasia, delayed eruption of permanent teeth, and fused roots [10].

Dental agenesis affects the growth of the jaws and leads to deficient growth of the alveolar bone. Patients present bimaxillary retrusion with respect to the anterior cranial base, negative overjet, a decreased vertical dimension that favors antero-rotation of the jaw and a tendency to skeletal class III, increasing the projection of the chin [12].

The functional and aesthetic rehabilitation of these patients is a challenge for dentists, so a multidisciplinary team is necessary. Early treatment during childhood is essential to solve the problem of multiple missing teeth and enhance the growth of the jaws to achieve better oral function and facial aesthetics. Initial dental treatment should focus on preventing cavities, restoring teeth with alterations in shape, replacing absent pieces, controlling the position of existing teeth and preventing or correcting malocclusions [14]. The first rehabilitations are usually made with removable complete or partial prostheses supported on the existing teeth previously restored aesthetically. Often these prostheses carry expansion screws to accompany maxillomandibular growth. As the patient grows, there is a need for orthopedic and/or orthodontic treatments for the management of the available space, leveling and aligning the teeth, and for orthopedic correction of the dentoskeletal malocclusion or jaw deformities.

In addition to a multidisciplinary group of dentists, the simultaneous participation of psychologists and speech therapists, who increase the patient’s self-esteem, is necessary to achieve self-acceptance and social integration [14–16].

Since patients with ED require orthodontic and/or dentofacial orthopedic treatment for functional and aesthetic rehabilitation, we formulated the following question*:* Which orthodontic and dentofacial orthopedic treatments have been carried out in patients with ectodermal dysplasia to facilitate functional and aesthetic rehabilitation?

The question was organized using the PICO strategy [17]:

Q: patients with ectodermal dysplasia.

I: orthodontic and dentofacial orthopedic treatments carried out in patients with ectodermal dysplasia.

C: not applicable.

O: orthopedic correction of the dentoskeletal malocclusion and/or management of the available space to facilitate functional and aesthetic rehabilitation in patients with ectodermal dysplasia.

## Methods

The systematic review was conducted in accordance with the Preferred Reporting Items for Systematic Reviews and Meta-analysis (PRISMA) statement [18]. The review was registered as CRD42021288030 in the Centre for Reviews and Dissemination, University of York, York, United Kingdom.

### Eligibility criteria

According to the selection criteria, the review included articles that described patients with any type of ED who received orthodontic or dentofacial orthopedic treatment to facilitate functional and aesthetic oral rehabilitation. Studies in animals, articles describing treatments for healthy patients or with other syndromes, studies describing aesthetic and/or functional treatments in patients with ED, but not requiring orthodontic or orthopedic correction, were excluded. Articles which stated that patient had been treated with orthodontics or dentofacial orthopedics, but without describing the procedure performed, were excluded.

### Information sources, search strategy and selection process

An exhaustive search of original articles was carried out in PubMed, Web of Science, Scopus, Scielo, LILACS, EBSCOhost and Embase. The search was conducted until 6 January 2022 and was not restricted by language or year of publication.

The keywords used in the search corresponded to the MeSH terms: "ectodermal dysplasia", "orthodontic", "orthopedic", and "treatment". The Boolean operators "OR" and "AND" were used to join keywords and establish a single search equation. This was used in the same way in the various databases to obtain the largest number of references. The equation was: ((ectodermal dysplasia) AND (orthodontic OR orthopedic) AND (treatment)). In addition, we manually reviewed the references of all selected articles to check whether there were any relevant references not found during the initial search that could be included.

### Data collection and quality assessment

We used a three-stage selection process. Firstly, we reviewed the title. Second, we read the abstract, but if this did not provide enough information to decide on inclusion, the entire article was reviewed. Third, the full text of the article was read. Two authors (MC and AJO) independently carried out the three stages and resolved any disagreement through discussion, until a consensus was reached. If disagreements persisted the authors consulted a third author (CS) who helped establish consensus. Data from the articles included were extracted by the same two authors and included authors, year of publication, country where the study was conducted, language, sample size, type of disease, sex, age of participants at the beginning of treatment, duration of treatment, type of treatment, dentition in which the treatment was performed, orthodontic/orthopedic treatment success, final rehabilitation, pattern of tooth agenesis, missing teeth, disturbances of the teeth, jaws affected, radiologic studies and cephalometric data.

The quality of the studies was assessed using the Joanna Briggs Institute Quality Assessment Scale, University of Adelaide, for case series and case reports [19] Quality was assessed by completing 8 items for each case report and 10 items for each case series. Items were answered "yes", "no", "unclear", or "not applicable" (Additional file 1: Tables 1 and 2). For each item of each article, the percentage of the answers "yes", "no", "not clear", or "not applicable" was calculated as was the maximum and minimum percentages of "yes" and "no" and their corresponding medians of all articles (Table 1). We set the median value of the percentage of 'Yes' as a limit to determine whether a study was of quality.

**Table 1:** Classification of case reports and case series according to the Joanna Briggs Institute critical appraisal checklist

Type of study	References	Yes		No		Unclear		Not applicable
		n^*^/N^†^	%	n^*^/N^†^	%	n^*^/N^†^	%	n^‡^
Case report	Karwetzky et al*. * [41]	8/8	100.0%	–	–	–	–	–
	Waggoner et al*.* [45]	7/8	87.5%	–	–	1/8	12.5%	–
	Itthagarun et al*.* [46]	8/8	100.0%	–	–	–	–	–
	Köymen et al*.* [64]	7/8	87.5%	–	–	1/8	12.5%	–
	Suri et al*.* [47] ^#^	6/8	75.0%	1/8	12.5%	1/8	12.5%	–
	Yenisey et al*.* [48]	7/8	87.5%	–	–	1/8	12.5%	–
	Gruber et al*.* [49]	7/8	87.5%	1/8	12.5%	–	–	–
	Altug-Atac et al*.* [50]	7/8	87.5%	–	–	1/8	12.5%	–
	Ioannidou-Marathiotou et al*.* [51]	8/8	100.0%	–	–	–	–	–
	Van Sickels et al*.* [44]^#^	6/8	75.0%	1/8	12.5%	1/8	12.5%	–
	Akgun et al*.* [52] ^#^	6/8	75.0%	1/8	12.5%	1/8	12.5%	–
	Machorowska-Pieniążek et al*.* [53] ^#^	6/8	75.0%	–	–	2/8	25.0%	–
	Oblack et al*.* [66]	7/8	87.5%	–	–	1/8	12.5%	–
	Priya et al*.* [54]	7/8	87.5%	–	–	1/8	12.5%	–
	Fraiz et al*.* [55] ^#^	6/8	75.0%	1/8	12.5%	1/8	12.5%	–
	Shah et al*.* [43]	7/8	87.5%	–	–	1/8	12.5%	–
	Suja et al*.* [56]	7/8	87.5%	–	–	1/8	12.5%	–
	Bergendal et al*.* [57]	7/8	87.5%	–	–	1/8	12.5%	–
	Knobloch et al*.* [59]	7/8	87.5%	1/8	12.5%	–	–	–
	Celli et al*.* [15]	7/8	87.5%	–	–	1/8	12.5%	–
	Kuźniarski et al*.* [16]	8/8	100.0%	–	–	–	–	–
	Yajing et al*.* [61]	7/8	87.5%	1/8	12.5%	–	–	–
	Ierardo et al*.* [62]	7/8	87.5%	1/8	12.5%	–	–	–
	Szemraj-Folmer et al*.* [63]	7/8	87.5%	1/8	12.5%	–	–	–
	Wimalarathna et al*.* [14]	7/8	87.5%	–	–	1/8	–	–
	Gonzaga Luiz et al*.* [42]	8/8	100.0%	–	–	–	–	–
	MAXIMUM	8	100.0%	1	12.5%	–	–	–
	MINIMUM	6	75.0%	0	0	–	–	–
	MEDIAN	7	87.5%	0	0	–	–	–
Case series	Fotso. et al. [65] ^#^	6/10	60.0%	4/10	40.0%	–	–	–
	Kościelska et al*.* [58]	8/10	80.0%	2/10	20.0%	–	–	–
	Schanbl et al*.* [60]	8/10	80.0%	2/10	20.0%	–	–	–
	MAXIMUM	8	80.0%	4	40.0%	–	–	–
	MINIMUM	6	60.0%	2	20.0%	–	–	–
	MEDIAN	8	80.0%	2	20.0%	–	–	–

^*^The number of Joanna Briggs Institute checklist sub-items for each case report, case series evaluated as “Yes,” “No,” and “Unclear”

^†^Total number of sub-items applicable to the case report/case series

^‡^Number of sub-items not applicable to the case report/case series

^#^Case report or case series whose percentage of “Yes” does not exceed the median value

In addition, for each item, the percentage of answers "yes", "no", "not clear" and "not applicable" of the total of the articles of "case reports" (Table 2) and "case series" (Table 3) was calculated. Items for which the answer was "no" and "not clear" in > 50% of articles could introduce a bias in the interpretation of the results [20].

**Table 2:** Classification of case reports according to the Joanna Briggs Institute critical appraisal checklist sub-items

Item No	Checklist item description	Yes		No		Unclear		Not applicable
		n^*^/N^†^	%	n^*^/N^†^	%	n^*^/N^†^	%	n^‡^
1	Were patient’s demographic characteristics clearly described?	26/26	100.0%	–	–	–	–	–
2	Was the patient’s history clearly described and presented as a timeline?	18/26	69.2%	8/26	30.8%	–	–	–
3	Was the current clinical condition of the patient on presentation clearly described?	26/26	100.0%	–	–	–	–	–
4	Were diagnostic tests or assessment methods and the results clearly described?	26/26	100.0%	–	–	–	–	–
5	Was the intervention(s) or treatment procedure(s) clearly described?	26/26	100.0%	–	–	–	–	–
6	Was the post-intervention clinical condition clearly described?	25/26	96.2%	1/26	3.8%	–	–	–
7	Were adverse events (harms) or unanticipated events identified and described?	10/26	38.5%	1/26	3.8%	15/26	57.7%^$^	–
8	Does the case report provide takeaway lessons?	26/26	100.0%	–	–	–	–	–

^*^The number of case reports for each Joanna Briggs Institute checklist sub-item evaluated as “Yes,” “No,” and “Unclear”

^†^Total number of case reports

^‡^Number of case reports that are not applicable

^§^The percentage of studies rated “No” and “Unclear” > 50%

**Table 3:** Classification of case series according to the Joanna Briggs Institute critical appraisal checklist sub-items

Item No	Checklist item description	Yes		No		Unclear		Not applicable
		n^*^/N^†^	%	n^*^/N^†^	%	n^*^/N^†^	%	n^‡^
1	Were there clear criteria for inclusion in the case series?	3/3	100.0%	–	–	–	–	–
2	Was the condition measured in a standard, reliable way for all participants included in the case series?	2/3	66.7%	1/3	33.3%	–	–	–
3	Were valid methods used for identification of the condition for all participants included in the case series?	2/3	66.7%	1/3	33.3%	–	–	–
4	Did the case series have consecutive inclusion of participants?	–	–	3/3	100.0%^$^	–	–	–
5	Did the case series have complete inclusion of participants?	3/3	100.0%	–	–	–	–	–
6	Was there clear reporting of the demographics of the participants in the study?	3/3	100.0%	–	–	–	–	–
7	Was there clear reporting of clinical information of the participants?	3/3	100.0%	–	–	–	–	–
8	Were the outcomes or follow up results of cases clearly reported?	3/3	100.0%	–	–	–	–	–
9	Was there clear reporting of the presenting site(s)/clinic(s) demographic information?	3/3	100.0%	–	–	–	–	–
10	Was statistical analysis appropriate?	–	–	3/3	100.0%^$^	–	–	–

^*^Number of case series for each Joanna Briggs Institute checklist sub-item evaluated as “Yes,” “No,” and “Unclear”

^†^Total number of case series

^‡^Number of case series that are not applicable

^§^The percentage of studies rated “No” and “Unclear” > 50%

## Results

### Selection of studies

**Fig. 1:**
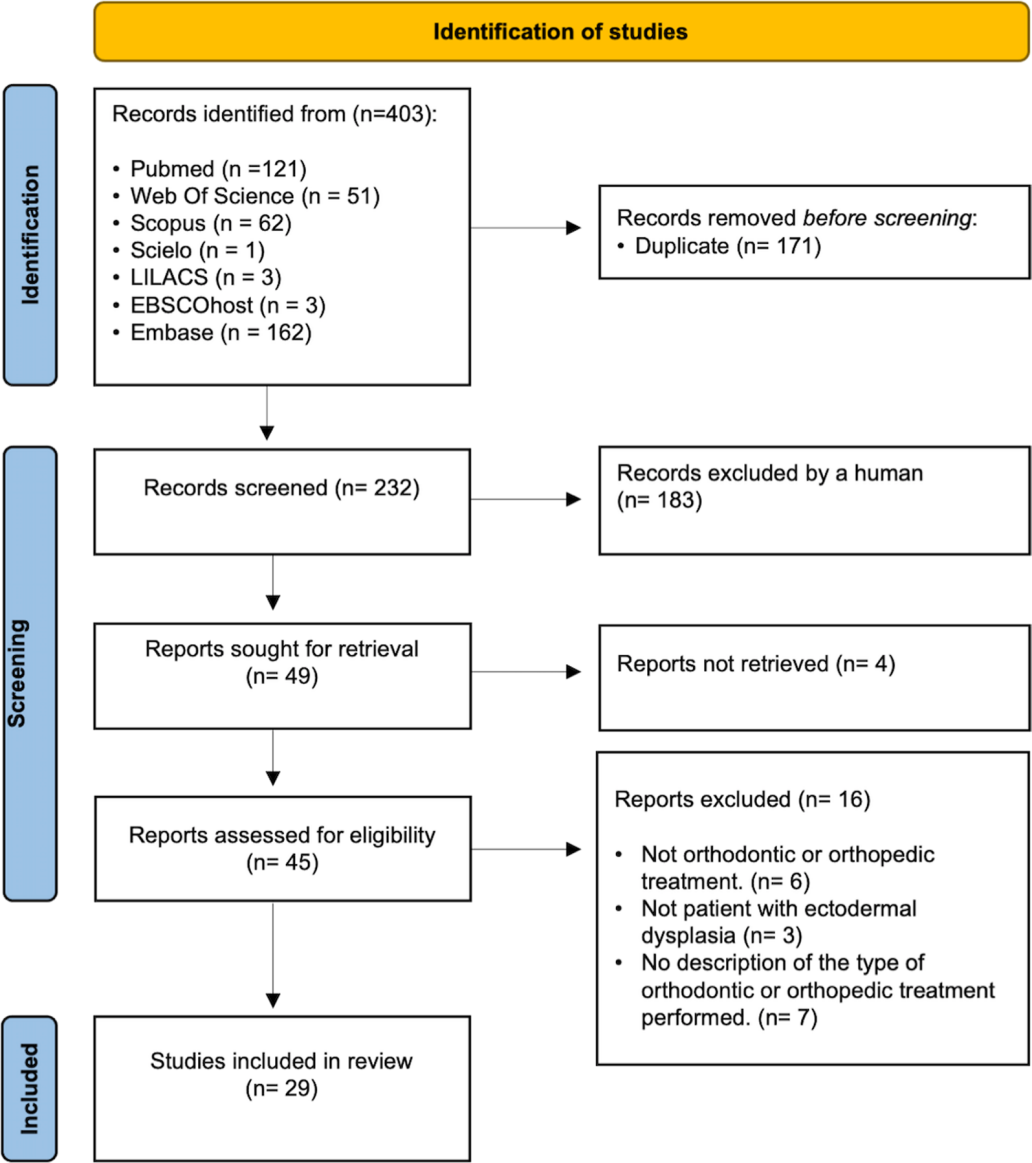
Flow diagram for search strategy

The initial search yielded 403 references. After deleting duplicate articles, 232 references remained. It was essential to read the abstracts of the 232 articles because the titles did not show, in most cases, the type of treatment. We discarded 183 references. Of the remaining 49, four [21–24] were eliminated due to the impossibility of obtaining the full text, even when requested from the authors. Six studies [25–30] were excluded because, although the summary indicated it, no orthodontic or dentofacial orthopedic treatment was performed; three [31–33] because it was another syndrome, the type of syndrome was not specified or it was in healthy patients; seven [34–40] because, although orthodontics or orthopedics were carried out in patients with ED, the type of treatment was not described. Twenty-nine articles met the inclusion criteria. (Fig. 1).

Of the 29 studies, the oldest was published in 1974 [41] and the most recent in 2021 [42]. Twenty-five of the references found were in English [14–16, 42–63], and one each in German [39], Turkish [64], French [65] and Slovenian [66].

Twenty-six articles were case reports [14–16, 41–57, 59, 61–64, 66] and three were case series [58, 60, 65].

### Quality of studies

The median percentage of "Yes" answers for case reports was 87.5%, and 22 studies were considered of quality [14–16, 41–43, 45, 46, 48–51, 54, 56, 57, 59, 61–64, 66], while five did not meet the quality criterion [44, 47, 52, 53, 55]. The median value for case series was 80%: two articles were considered of quality [58, 60], and one was not [65] (Table 1).

Item 7 "Adverse events or unforeseen treatment events " had a percentage of "no" or "unclear" responses of > 50% for case reports (Table 2) and item 4 "selection of participants" and 10 "appropriate statistical analysis" in case series (Table 3).

### Syntheses of studies

Twenty-five patients (6 female and 19 male) were included in the 23 articles: 21 articles only included one patient [14–16, 41–43, 45, 46, 48–51, 54, 56, 57, 59, 61–64, 66] and 2 [58, 60] included 2 patients. The age of patients included ranged from 34 months [45] to 24 years [56] (Table 4).

**Table 4:** Characteristics of case reports and case series included

Reference/Country/Language	Nº patients Type of disease Sex^*^ (M/F) Age^**^ (I/F)	Orthodontic and/or dentofacial orthopedic treatment Treatment of permanent or deciduous dentition Success in orthodontic and/or dentofacial orthopedic treatment Esthetic and functional rehabilitation	Pattern of tooth agenesis Missing teeth Disturbances of the teeth Jaws affected	Radiologic study Cephalometric study
Karwetzky et al*.* [41]/Germany/German	N = 1 ED Sex: M Age: I: 8 y F: 2 y	Orthopedic: U-Bar activator type I to stimulate maxillary growth Orthodontic: canine bands to increase intercanine width Treatment of permanent canines Outcomes: intercanine distance increased to 18–20 mm (7 months) Upper and lower removable prostheses	Mandibular anodontia/Maxillary oligodontia All permanent teeth missing, except two maxillary canines No disturbances in the two erupted canines Maxilla and mandible affected	No OPG No cephalometric study
Waggoner et al*.* [45]/New York, USA/English	N = 1 HED Sex: M Age: I: 34 m F: 7 weeks	Orthodontic: Brackets for dental alignment to close 6 mm wide inter-incisive diastema, twice due to recurrence. Two brackets, rectangular wire and elastics changed at 3-week intervals Treatment of deciduous dentition Successful treatment: closed diastema and at 8 months X-ray showed no radicular resorption Upper and lower removable prostheses	Mandibular oligodontia/Maxillary oligodontia All permanent teeth were missing, except two maxillary central incisors and mandibular first molars Conically-shaped primary maxillary central incisors Maxilla and mandible affected	OPG. Mandibular occlusal X-ray No cephalometric study
Itthagarun et al*.* [46]/Hong Kong/English	N = 1 HED Sex: F Age: I: 7 y F: 6 m	Orthodontic: (1) maxillary removable orthodontic appliance with finger spring to close the space between the inter-incisive diastema; (2) fixed appliance with open coil spring, due to loss of removable appliance Treatment of permanent dentition Successful treatment: closed diastema Upper and lower removable prosthesis	Mandibular oligodontia/Maxillary oligodontia Missing deciduous teeth: 62, 64, 71, 72, 81, 82 Missing permanent teeth: 11, 12, 13, 14, 16, 17, 22, 23, 24, 25, 26, 27, 31,32, 33, 35, 36, 37, 41, 42, 43, 45, 46, 47 Conical teeth: 51, 52, 53, 61, 63. 61 showed a shorter root compared with its antimere Maxilla and mandible affected	OPG No cephalometric study
Köymen et al*.* [64]/Turkey/Turkish	N = 1 ED Sex: M Age: I: 16 y F: -	Orthodontic: brackets to close inter-incisor diastema Treatment in permanent dentition Successful treatment: closed diastema Upper and lower removable prosthesis	Mandibular oligodontia/Maxillary hypodontia Missing permanent teeth: 12, 13, 22, 23, 31, 32, 33, 41, 42, 43 No disturbances of the teeth Maxilla and mandible affected	OPG No cephalometric study
Yenisey et al*.* [48]/Turkey/English	N = 1 HED Sex: M Age: I: 10 y F: 7 m	Orthodontic: a button was placed on the retained tooth in the palate after the mucosa was opened surgically. Another button was placed on the upper removable prosthesis. Elastics were attached between the buttons to apply a 60-g force Treatment of permanent dentition Successful treatment: closed diastema Upper and lower removable prosthesis	Mandibular anodontia/Maxillary oligodontia All mandibular permanent teeth were missing. Radiographic examination showed a conical impacted tooth in the middle of the right anterior maxilla, the rest of the teeth were missing There were two conical, hypoplastic anterior teeth in the left maxilla Maxilla and mandible affected	OPG No cephalometric study
Gruber et al*.* [49]/New York/English	N = 1 HED Sex: M Age: Phase 1: I: 7 y F: 7 m Phase 2: I: 8 y F: 3 y	Phase 1: Orthopedic treatment: palatal expander and protraction headgear—retainers with spring Orthodontic diagnosis: anterior crossbite, severe class III malocclusion, retrusion maxillary, deficient maxillary and mandibular height Goal: to enhance alveolar growth and establish a class I occlusion Outcomes: overjet 3–4 mm Phase 2: protraction headgear Goal: To correct rapid growth of the mandible and a current edge-to-edge anterior bite relationship Negative sequelae: gingival recession of the vestibular aspect of 72 Outcomes: Class I relationship and 4 mm overjet Treatment of deciduous and permanent dentition No prosthetic treatment has been performed so far	Mandibular and maxillary oligodontia Missing permanent teeth: 12, 13, 15, 22, 23, 24, 25, 31, 33, 34, 35, 37, 41, 42, 44, 45, 47 Deciduous teeth: 20 conical shaped microdontic teeth, amelogenesis imperfecta. The roots were unusually long Permanent teeth: 11 hypocalcified, microdontic and conically shaped Maxilla and mandible affected	OPG (5 ½, 8 and 10 years) Lateral skull X-ray (6, 7 ¾ and 10 years) No cephalometric study
Altug-Atac et al*.* [50]/Turkish/English	N = 1 EDW Sex: M Age: Phase I: I: 6 y. and 4 m F: 6 m Phase II: I: 9 y. and 3 m F: 6 m. + 6 m. for retention Phase III: I: 10 y. and 3 m F: 21 m	Phase I: Removable tongue shield was inserted to rehabilitate tongue Phase II: Class II activator was applied to stimulate mandibular growth and eliminate the 12.5-mm overjet Phase III: Orthodontic treatment. Dental alignment with fixed brackets. Intermaxillary class II elastics to achieve and maintain the skeletal class I relationship. Coil springs to close the spaces Treatment of deciduous and permanent dentition Success achieved in all phases of treatment No prosthetic treatment indicated	Mandibular hypodontia Missing permanent teeth: 41, 42, 31 53, 63, 73, 83, 12, 22, 31, 32, 41, 42 were conically shaped Class II, division I malocclusion. Retrognathic mandible	OPG (before and after treatment) 4 cephalometric studies Phase II: Skeletal class II (ss-n-sm: 7.71°) severe hyperdivergent growth pattern (NSL/ML: 39.82°) (ILs/NL: 114.4º and ILi/ML: 92.4º) End of treatment: ss-n-sm: 6.06° NSL/ML: 43.36° ILs/NL: 92.71° ILi/ML: 85.42°
Ioannidou-Marathiotou et al*.* [51]/Greece/English	N = 1 HED Sex: M Age: I: 10 years F: 6 y. and 8 m	Orthopedic: removable partial prothesis with palatal expansion screw. Goal: to enhance the transverse growth of the maxilla Orthodontic: brackets with a segmental arch wire and elastic chain for dental alignment. Goal: to close the midline diastema Treatment in permanent and deciduous dentition There was an increase in the transverse dimensions of the upper dental arch Removable upper and lower prostheses	Mandibular anodontia, maxillary oligodontia All mandibular deciduous and permanent teeth were missing. In maxillary arch 53, 63, 11, 16, 21, 26 were present 53, 63, 11, 21 showed conical shaped Mandible and maxilla affected	OPG (before and after treatment) 2 cephalometric studies 10 years: concave skeletal profile (ANB: − 6.7°, retrognathic maxilla (SNA: 72.7°) decreased lower face height. Midline diastema of 5 mm 16 years and 8 months: face had normal vertical dimensions and the anteroposterior skeletal discrepancy had improved. (SNA: 73.8º; ANB: − 0.4º)
Oblack et al*.* [66]/Slovenia/Slovene	N = 1 ED Sex: M Age: I: 12 y F: –	Orthodontic: brackets to dental alignment and maintenance of space Treatment of permanent and deciduous dentition Orthodontic treatment allowed rehabilitation with 4 maxillary implants, crowns and bridges	Maxillary and mandibular oligodontia Missing permanent teeth: 12, 13, 14, 15, 22, 24, 25, 31, 32, 34, 35, 41, 42, 44 53, 11, 21, 23, 33, 43 conical shaped Maxilla and mandible affected	OPG No cephalometric study
Priya et al*.* [54]/India/English	N = 1 HED Sex: F Age: I: 22 y F: –	Orthodontic: brackets for dental alignment Treatment of permanent and deciduous dentition Orthodontic treatment allowed rehabilitation with 2 maxillary and 1 mandibular implants, crowns and bridges, to recover the reduced vertical dimension	Maxillary and mandibular hypodontia Missing permanent teeth: 15, 12, 22, 27, 38, 45 Generalized Microdontia. Canines had conical shaped. Molars appeared malformed with obliterated occlusal tables Maxilla and mandible affected	OPG No cephalometric study
Shah et al*.* [43]/India/English	N = 1 XHED Sex: M Age: I: 21 y F: 6 m	Orthodontic: brackets for erupted canine tooth retained in the palate and dental alignment Treatment of permanent dentition The tooth retained was exposed Rehabilitation was done with 3 mandibular implants, crowns and bridges	Maxillary and mandibular oligodontia Missing permanent teeth: 14, 16, 17, 22, 24, 25, 27, 31, 32, 33, 36, 37, 41, 42, 43, 46 Two impacted teeth in the right maxillary canine region and one in the left maxillary canine region Maxilla and mandible affected	OPG No cephalometric study
Suja et al*.* [56]/India/English	N = 1 HED Sex: F Age: I: 24 y F: -	Orthodontic: brackets for dental leveling and aligning Treatment of permanent and deciduous dentition Orthodontic treatment allowed rehabilitation with crowns and fixed prothesis in permanent and primary retained teeth	Mandibular and maxillary oligodontia Missing permanent teeth: 12, 14, 15, 17, 22, 24, 25, 27, 31, 32, 33, 37, 41, 42, 43, 47 Anterior teeth were conically shaped Maxilla and mandible affected	OPG No cephalometric study
Bergendal et al*.* [57]/Sweden/English	N = 1 ED Sex: M Age: I: 21 y F: 12 mo	Orthodontic: dental leveling and aligning Treatment of permanent and deciduous dentition At 6 y and 4 mo placement of two mandibular implants to retain overdenture. Maxillary removable denture At 19 y and 10 mo. Two additional mandibular implants to support fixed complete prosthesis After orthodontic treatment two maxillary tooth supported prostheses, preserving a small midline diastema	Mandibular anodontia and maxillary oligodontia All deciduous teeth were missing except two maxillary incisors and canines All permanent teeth were missing except 11, 21, 16 and 26 Deciduous teeth were conically shaped. Permanent teeth were malformed Maxilla and mandible affected	OPG (5y and 6mo, 7y, 33y) Cephalometric studies: 7y and 3mo: Normal maxilla and normal sagittal and vertical relation with the mandible 10y and 4mo: slight class III with ANB -1° and ANPg -2° 20y and 4mo: slight class III 32y and 5mo: the angulated abutments of mandibular prosthesis compensated for the slight class III and the profile looked similar to a class I
Kościelska et al*.* [58]/Poland/English	N = 2 P2 ED Sex: M Age: I: 5 y F: - P3 ED Sex: M Age: I: 12 y F: -	P2 Orthopedic: maxillary and mandibular prostheses with Fisher screws to expand Treatment of deciduous dentition Success in esthetic and functional rehabilitation with the removable prosthesis with screws P3 Orthopedic: acrylic denture for maxilla and mandible with Fisher screws Treatment of permanent and deciduous dentition Success in esthetic and functional rehabilitation with the removable prosthesis with screws	P2 Mandibular oligodontia/Maxillary oligodontia All deciduous teeth were missing except 55, 65, 85 55, 65, 85 microdontics Maxilla and mandible affected P3 Mandibular oligodontia/Maxillary oligodontia Missing permanent teeth: 12, 13, 14, 17, 22,23, 24, 25, 31, 32, 33, 34, 35, 37, 41, 42, 43, 45, 47 No disturbance in the erupted teeth Maxilla and mandible affected	P2 No OPG No cephalometric study P3 OPG No cephalometric study
Knobloch et al*.* [59]/Canada/English	N = 1 HED Sex: M Age: Phase I: I: 11 y F: – Phase II: I: 18–9 y F: 2–3 y	Phase I (11 y.): Presurgical orthodontics with brackets. Retention with Hawley plate Goal: To address the impacted left posterior segment and the midline diastema and consolidate the spaces of anterior maxillary teeth Orthodontic treatment allowed consolidation of anterior maxillary spaces, but the left impacted teeth could not be repositioned, and the midline diastema could not be totally closed Phase II: Fixed postsurgical orthodontics. (The orthognathic surgery was Le Fort type I to correct maxillary AP hypoplasia at end of growth) Goal: to control tooth position and aid the surgeon in maintaining proper maxillary stability Orthodontic phase II was successful Esthetic functional rehabilitation: 10 y. Two mandibular anterior implants to support overdenture 20 y. 3 additional implants in anterior mandible and 2 implants in posterior maxilla 21 y. In maxilla, crowns and removable prosthesis tooth-implant supported. In mandible, fixed prosthesis implant supported	Mandibular oligodontia/Maxillary oligodontia All deciduous teeth were missing except 65. Permanent missing teeth: 13, 14, 15, 17, 23, 24, 25, 27, 31, 32, 33, 34, 35, 37, 41, 42, 43, 44, 45, 47 11, 12, 21, 22 were conically shaped. 16 were dysmorphic Maxilla and mandible affected	OPG. (10, 18–19, 20, 21 y.) Lateral skull X-ray (17 y.) Cephalometric study: maxillary AP hypoplasia
Celli et al*.* [15]/Italy/English	N = 1 HED Sex: M Age: I: 6 y F: 4 y	Phase 1: Maxilla and mandible: Removable orthopedic/prosthesis with repositioning of teeth and expansion screw Phase 2: Maxilla: maxillary rapid expander partially removable and partially fixed. The device repositioned the anterior missing teeth Mandible: Fixed orthopedics with reposition of teeth and telescopic screw Treatment of permanent and deciduous dentition Success in dentofacial orthopedic treatment in both phases Rehabilitation was with the same prosthesis	Maxillary hypodontia and Mandibular oligodontia Deciduous missing teeth: 51, 52, 61, 62, 71, 72, 81, 82 Permanent missing teeth: 11, 12, 15, 21, 22, 31, 32, 35, 37, 41, 42, 45, 47 Deciduous canines were conically shaped Maxilla and mandible affected	OPG Cephalometric study (Steiner and Rickets) Skeletal class I with a trend to class III Maxillary and mandibular retrognathism Vertical hypodivergent skeletal pattern Class I molar and canine Overjet and overbite could not be estimated because all front teeth were absent
Kuźniarski et al*.* [16]/Poland/English	N = 1 ED Sex: M Age: I: 3 y F: -	Orthopedic: removable plate with reposition of teeth and Fisher expansion screws in the maxilla and mandible Treatment in deciduous dentition No information on expansion success. The diastema was not treated Rehabilitation was with the same removable prosthesis	No information on permanent teeth Deciduous missing teeth: 52, 53, 54, 62, 63, 64, 71, 72, 73, 74, 75, 81, 82, 83, 84, 85 Deciduous incisors with a conical, strongly sharpened shape Maxilla and mandible affected	No OPG No cephalometric study
Schanbl et al*.* [60]/Austria/English	N = 2 P1 XHED Sex: M Age: I: 10 y F: - P2 XHED Sex: M Age: I: 17 y F: –	P1 Orthodontic: the diastemas between the anterior maxillary teeth were closed by a fixed orthodontic device Treatment in deciduous dentition The diastemas were closed At 10 y. rehabilitation was made with a mandibular tooth-supported overdenture and a maxillary removable two-implant-retained prosthesis P2 Orthodontic: pre-post orthodontic gap closure between 21 and 22 by means of a spring attached to the removable denture Treatment in permanent dentition The gap was closed Rehabilitation was carried out with 4 maxillary and 4 mandibular implants, crowns, and partial removable dentures tooth and implant attached	P1 Mandibular oligodontia/Maxillary oligodontia Deciduous missing teeth: 52, 54, 55, 62, 64, 65, 71, 72, 73, 74, 75, 81, 82, 83, 84, 85 Permanent missing teeth: 12, 13, 14, 15, 16, 17, 22, 23, 24, 25, 26, 27, 31, 32, 34, 35, 37, 41, 42, 44, 45, 46, 47 All anterior teeth erupted were conically shaped Maxilla and mandible affected P2 Mandibular oligodontia/Maxillary oligodontia Permanent missing teeth: 11, 12, 14, 15, 16, 17, 23, 24, 25, 26, 27, 31, 32, 33, 34, 35, 37, 41, 42, 43, 44, 45, 47 All erupted anterior teeth were conically shaped Maxilla and mandible affected	P1 OPG (6, 10 y.) No cephalometric study P2 OPG (17, 23 y.) No cephalometric study
Ierardo et al*.* [62]/Italy/English	N = 1 HED Sex: M Age: I: 8 y F: 12 days activation + 6 m. retention	Orthopedic: hybrid modified rapid palatal expander with braces in 6 + 6 and two mini-screws in the anterior palatal region Treatment of permanent and deciduous dentition Successful resolution of the transverse contraction Definitive treatment not indicated	Mandibular oligodontia/Maxillary oligodontia Deciduous missing teeth: 52, 54, 62, 64, 71, 72, 73, 74, 81, 82, 83, 84 Permanent missing teeth: 12, 13, 14, 15, 16, 17, 22, 23, 24, 25, 26, 27, 31, 32, 33, 34, 35, 37, 41, 42, 43, 44, 45, 47 All erupted anterior teeth were conically shaped Maxilla and mandible affected. Hypotrophy of both jaws	OPG Occlusal X-ray. To verify the diastases of the median palatal suture CBCT to verify the quantity of available bone and space for correct insertion of miniscrews Cephalometric study Class I with a light trend to skeletal class III Hypo-divergence Counterclockwise growth Reduced anterior facial height
Szemraj-Folmer et al*. *[63]/Poland/English	N = 1 ED Sex: F Age: Phase I I: 8 y F: 2 y Phase II I: 11 y F: 58 m	Phase 1. Removable orthodontic appliance for distalization of the maxillary right first molar prior to autologous transplantation Phase 2: Fixed orthodontics for dental alignment, space maintenance and occlusion after autologous transplantation The objectives of orthodontic treatment were achieved: orthodontic alignment of the teeth (including the transplanted premolars) closing spaces at the donor sites reducing the space between the mandibular left first molar and canine The end of rehabilitation treatment not explained	Maxillary hypodontia/mandibular hypodontia Missing teeth 12, 22, 23, 24, 25, 34, 35, No disturbances of the teeth Maxilla and mandible affected	OPG (8, 15 and 16 y.) No cephalometric study
Yajing Liu et al*. *[61] /China/English	N = 1 ED Sex: F Age: I: 17 y F: 12 m	Orthodontic: brackets for dental alignment and inter-incisor diastema closure Treatment of permanent dentition Orthodontic treatment achieved closure of the diastema Definitive rehabilitation was carried out in maxilla with 2 esthetic crowns on central incisors and 2 partial fixed prostheses 6-implant-supported. In mandible with a complete prosthesis supported by 6 implants	Mandibular oligodontia/Maxillary oligodontia Permanent missing teeth: 12, 13, 14, 15, 17, 22, 23, 24, 25, 27, 31, 32, 33, 34, 35, 37, 41, 42, 43, 44, 45, 47 Erupted maxillary and mandibular anterior teeth were conically shaped Maxilla and mandible affected	OPG No cephalometric study
Wimalarathna et al*. *[14]/Sri Lanka/English	N = 1 HED Sex: M Age: I: 11 y F: 5 y	Orthodontic: brackets for dental alignment and space maintenance The objective of orthodontic treatment was achieved Treatment in permanent and deciduous dentition Final rehabilitation was achieved with esthetic crowns	Mandibular hypodontia/maxillary hypodontia Permanent missing teeth: 12, 15, 22, 23, 31, 41 53, 62, 63, 73, 81, 83, 32, 42 conically shaped. First lower molars with taurodontism Maxilla and mandible affected	OPG No cephalometric study
Gonzaga Luiz et al*. *[42]/Brazil/English	N = 1 ED Sex: F Age: I: 19 y F: 8 m	Orthodontic: DSD (digital smile design) aligners program-based Treatment in permanent and deciduous dentition Success of orthodontic treatment was partial, as the tooth alignment was achieved but not the extrusion needed to reestablish the vertical dimension Rehabilitation was made with esthetic crowns on natural tooth and implants (2 in maxilla and 2 in mandible)	Mandibular oligodontia/maxillary hypodontia Permanent missing teeth: 12, 13, 22, 23, 31, 32, 37, 41, 42, 43, 47 Erupted maxillary and mandibular anterior teeth were conically shaped Maxilla and mandible affected	OPG CBCT Cephalometric study, before and after orthodontic treatment, but not described

*ED* Ectodermal dysplasia (type not specified), *EDW* Ectodermal dysplasia (Wiktop syndrome), *HED* Hypohidrotic ectodermal dysplasia, *XHED* X-linked hypohidrotic ectodermal dysplasia, *OPG* Orthopantomography, *CBCT* cone bone computer tomography

^*^Male (M) and Female (F)

^**^Age at the start of orthodontic treatment (I). Follow-up of orthodontic treatment (F). y: years. mo: months

Most patients had hypohidrotic and/or anhidrotic ED [14, 15, 43, 45, 46, 48, 49, 53, 56, 57, 59, 60, 62], specifically X-linked in two studies [43, 60]. In one study [50], the patient had Wiktop syndrome (ORPHA:2228; ICD-10: Q82.4; OMIM:189500). In the rest [16, 41, 42, 57, 58, 61, 63, 64, 66], the type of ED was not specified.

### Type of treatments

Diverse treatments were carried out.

- Fixed orthodontic appliances or simple acrylic plates designed for tooth movement including leveling and aligning, closing of diastema, retraction of impacted teeth in the dental arch.
  Four studies [45, 60(P1), 61, 64] used only fixed orthodontics to close the diastema between the anterosuperior teeth. In one [45], there was a recurrence, and the patient was treated again with fixed orthodontics. In others [46, 60(P2)], a removable acrylic resin plate with a spring was used to close the existing diastema. In one [46], after a recurrence due to the loss of the removable device, a fixed cemented device was chosen in the first molars, with an open coil spring to close the diastema again. In eight studies [14, 43, 48, 54, 56, 57, 63, 66], orthodontics was used to achieve a correct dental position through alignment with brackets. In two [43, 48], in addition to aligning the teeth, fixed appliances were used to traction a tooth retained in the jaw. In two studies [14, 66], in addition to alignment, spaces were maintained for future fixed prosthetic rehabilitation. One study [63] described two-phase orthodontic treatment, the first with removable orthodontics to obtain space to perform an autologous transplant and the second with brackets to align the teeth, maintain the spaces and adjust the occlusion.
- Clear aligners.
  One article [42] included the use of aligners designed with Digital Smile Design software to position the teeth.
- Fixed and/or removable appliances for the correction of skeletal and/or dentoalevolar relationships.
  Some patients underwent removable prosthetic-orthopedic apparatus with expansion screws [15, 16, 58] or telescopic devices [15] to replace missing teeth while stimulating or accompanying the growth of the jaws. Another patient [51] presented a concave profile (ANB: − 6.7º), with a retrognathic maxilla (SNA: 72.7º) and a decreased lower facial height, who initially needed an upper prosthesis with an expansion screw and, later, fixed orthodontics with brackets to align the teeth. After orthopedic and orthodontic treatment, a face with normal vertical dimensions was observed and the anteroposterior skeletal discrepancy had improved (ANS: 73.8º; ANB: − 0.4º).
  One study [50] described a patient with Witkop syndrome who presented skeletal class II (ss-n-sm: 7.7º) with an increased overjet (12. 5 mm). A functional device was used to stimulate the growth of the jaw, achieving an ss-n-sm angle of 6.06º and an overjet of 3.51mm. Finally, brackets were used for dental alignment. Another study [41] used fixed orthodontics with bands in the canines to increase the intercanine width, after carrying out a first phase of orthopedics with a type I U-activator to stimulate the growth of the jaws. In another study [62], a hybrid maxillary expansion appliance was used, with dental anchorage in the first two upper molars and skeletal anchorage by means of two microscrews in the anterior area of the palate.
- Palatal expanders in combination with face masks for orthopedic traction of the maxilla.
  In one study [49], palatine disjunction was carried out and a facial mask was placed to improve the growth of the maxilla in the transverse and sagittal planes.
- Orthognathic surgery.
  In one study [59], the treatment began at 9 years of age, with two implants placed in the jaw in order to place an implant-supported prosthesis that would improve function and replace the missing teeth. At the age of 11, a fixed orthodontic treatment in the maxilla was carried out to align, close and consolidate spaces. Retention after orthodontic treatment consisted of a Hawley plate that incorporated the missing teeth. Once the patient's growth was complete (18–19 y.) they underwent orthognathic surgery (Lefort type I) and subsequent fixed orthodontics with braces.

### Treatment duration

The treatment duration varied from 7 weeks [45] to 10 years [15], depending on the purpose and type of treatment. Seven weeks were necessary to close an inter-incisor gap with fixed appliances [45].

In seven studies, treatment lasted for 4 to 12 months. Of these, 5 used fixed orthodontics: one [57] to align; two [46, 61] to close the inter-incisor gap and two [43, 48] to traction a tooth included in the maxilla and align. One study [42] used aligners and another [62] orthopedics to achieve rapid palatal disjunction.

Several studies had treatment periods of > 12 months: two carried out a first phase with orthopedics and a second with fixed orthodontics, lasting 2 years [41] and 3 years and 3 months [50]. In a study [63] in which treatment lasted 4 years and 10 months, orthodontics prior to an autotransplant and subsequent orthodontics were carried out. In another study treatment lasted five years [49] using first a disjunction and then a face mask. In two studies, treatment lasted seven years: in one there was an initial phase with an upper removable prosthesis with an expansion screw and then dental alignment with brackets [51] and, in a second there was fixed orthodontic treatment before and after a LeFort type I surgery [59]. The maximum treatment period was 10 years [15]: firstly, there was rehabilitation with a removable prosthesis with upper and lower expansion screws and in a second phase these were replaced by another upper prosthesis with an expansion screw and a lower one with a telescopic device. The remaining studies did not specify [14, 16, 54, 56, 58, 60, 64, 66] the treatment duration.

## Discussion

To our knowledge, this is the first systematic review of the different orthodontic and dentofacial orthopedic treatments carried out in patients with ED.

### Discussion of the methodology

As we reviewed orthodontic and dentofacial orthopedic treatments in patients with ED to facilitate aesthetic and functional rehabilitation, the type of studies found in the search were case series and case reports. These are observational studies and, therefore, the scientific evidence is not high. This is the main limitation of our study. Another limitation was the difference between the studies analyzed in the type of treatment performed, the age of participants, the follow-up time, and the provision of cephalometric data before and after treatment. The lack of effect measures in the studies did not allow a meta-analysis. Although randomized controlled trials have the highest level of evidence, they are very difficult to carry out in this type of patient, since they need individualized treatment to provide the function and aesthetics necessary for a normal quality of life.

To assess the quality of case series and case reports, most systematic reviews use the Newcastle–Ottawa Quality Scale (NOS), which is specific for case–control and cohort studies, but not for case series and case reports. Therefore, we opted to use the quality assessment scale of the Joanna Briggs Institute of the University of Adelaide for case series and case reports [19]. A "no" or "unclear" answer to any of the items causes a negative impact on the overall quality of the study analyzed. Therefore, studies that did not exceed or equal the percentage of the median "yes" were eliminated [44, 47, 52, 53, 55]. In addition, items with a "no", or "unclear" response rate of > 50%, which could have introduced bias in the evaluation of the articles, were item 7 "adverse events or unforeseen events of the treatment" in case reports and item 4 “selection of participants" and item 10 "appropriate statistical analysis" in the case series. These items, in our case, did not introduce bias since, in the case reports, the fact that an adverse effect is not described does not necessarily indicate that it may exist and, in the case series, there is no prior selection of participants, and neither is a statistical analysis essential.

### Discussion of results

Historically, the oral rehabilitation of patients with ED was carried out using partial or completely removable prostheses, whether mucous or dento-supported. Currently, when the number and position of the teeth is favorable, the treatment of choice is usually fixed prostheses on natural teeth with crowns or bridges [14, 43, 54, 56, 57, 61, 66] implant-supported prostheses [42, 43, 54, 61, 66] or implant-retained overdentures [57]. Orthodontic and dentofacial orthopedic treatment has been used, fundamentally, to facilitate subsequent prosthetic rehabilitation to restore the necessary function and aesthetics.

The most frequent orthodontic treatments were the closure of diastemas in the anterosuperior region [41–46, 60, 61, 64], the alignment or maintenance of spaces [14, 43, 48, 50, 54, 56, 57, 63, 66] and the traction of teeth included in the palate [43, 48], All are aimed at facilitating the placement of implants and/or subsequent prosthetic treatment. Although ED presents with a lack of maxillomandibular development, we found only four studies whose main objective was to improve maxillomandibular growth. Karwetzky et al. [41] placed a type I U-activator to stimulate maxillary development; Gruber et al. [49] used a palatine circuit breaker with a face mask to stimulate the growth of the maxilla horizontally and sagittally; Altug-Atac et al. [50] used a functional device to correct an open bite due to lingual habits plus an orthopedic device to stimulate mandibular growth, and Ierardo et al. [62] used a fixed breaker with bands in 6 + 6 and two microscrews on the anterior palate to achieve rapid disjunction. Other authors have used removable prostheses, necessary to replace the missing teeth, for orthopedic purposes, since these devices included expansion screws or telescopic screw, both in the maxilla and the jaw [15, 16, 51, 58].

The poor results obtained in terms of maxillomandibular growth could explain why only three studies [50, 51, 57] made cephalometric records of the follow-up of orthodontic and orthopedic treatments.

Almost all the treatments fulfilled the initial objective proposed, allowing aesthetic and functional rehabilitation. The duration of treatment varied widely and depended on the type of treatment. However, it was close to the duration of orthodontic or orthopedic treatment in a patient without ED, where the average treatment duration was 24.9 months [67]. This indicates that the tooth movement rate is probably not affected in the case of ED.

Patients with ED should be treated from a very early age with the aim of stimulating maxillomandibular bone growth, which is the main challenge in the aesthetic and functional rehabilitation of these patients. In addition, a multidisciplinary approach is necessary, where pediatric dentists, orthodontists, oral and maxillo-facial surgeons, prosthodontists, psychologists, speech therapists, etc. intervene with the aim of maintaining the existing dentition, improving aesthetics, speech, and masticatory efficiency, and acceptance by the patient and their environment, thus helping their psychological well-being and social integration.

Studies with a higher level of evidence, and more patients grouped by age, types of treatments and the use of cephalometric data before and after the procedures are needed to establish treatment protocols in these patients.

## Conclusions

Due to the nature of the problem, most studies reviewed had a low level of evidence. Most dentofacial orthopedic and orthodontic treatments described focused on correcting dental malpositions and asymmetries in the jaws and not on stimulating growth from an early age. Studies with more scientific evidence are needed to determine the best treatment for these patients.

Greater efforts should be made to stimulate maxillomandibular growth from birth in patients with ectodermal dysplasia to facilitate subsequent aesthetic-functional rehabilitation. A multidisciplinary approach would aid psychological well-being and social integration in these patients.

## Supplementary Information

**Additional file 1:** Responses to the Joanna Briggs institute critical assessment items for case reports and case series.

## Data Availability

All data generated or analyzed during this study are included in this published article and the supplementary information files.

## Abbreviations

ED: Ectodermal dysplasia (type not specified)
EDW: Ectodermal dysplasia (Wiktop Syndrome)
AED: Anhidrotic ectodermal dysplasia
HED: Hypohidrotic ectodermal dysplasia
XHED: X-linked hypohidrotic ectodermal dysplasia
