# Supplementary material for: Orthodontic and dentofacial orthopedic treatments in patients with ectodermal dysplasia: a systematic review
Source: Orphanet J Rare Dis. 2022 Oct 17;17:376. doi: 10.1186/s13023-022-02533-0 (PMC9575248; doi:10.1186/s13023-022-02533-0)
Supplement: Supplementary file 1 — Additional file 1: Responses to the Joanna Briggs institute critical assessment items for case reports and case series. [file 13023_2022_2533_MOESM1_ESM.pdf]

Supplementary Table 1. Responses to the Joanna Briggs Institute Critical assessment items for Case Reports.

CASE REPORTS								
Author(s)/year	Item No.							
	1	2	3	4	5	6	7	8
Karwetzky R *et al. (*1974) [41]	Yes	Yes	Yes	Yes	Yes	Yes	Yes	Yes
Waggoner WF *et al.* (1987) [45]	Yes	Yes	Yes	Yes	Yes	Yes	Unclear	Yes
Itthagarun A *et al.* (2000) [46]	Yes	Yes	Yes	Yes	Yes	Yes	Yes	Yes
Köymen G *et al.* (2003) [64]	Yes	Yes	Yes	Yes	Yes	Yes	Unclear	Yes
Suri S *et al.* (2004) [47]	Yes	No	Yes	Yes	Yes	Yes	Unclear	Yes
Yenisey M *et al.* (2004) [48]	Yes	Yes	Yes	Yes	Yes	Yes	Unclear	Yes
Gruber J *et al.* (2006) [49]	Yes	No	Yes	Yes	Yes	Yes	Yes	Yes
Altug- Atac AT *et al.* (2008) [50]	Yes	Yes	Yes	Yes	Yes	Yes	Unclear	Yes
Ioannidou-Marathiotou I *et al.* (2010) [51]	Yes	Yes	Yes	Yes	Yes	Yes	Yes	Yes
Van Sickels JE *et al.* (2010) [44]	Yes	No	Yes	Yes	Yes	Yes	Unclear	Yes
Akgun OM *et al.* (2010) [52]	Yes	No	Yes	Yes	Yes	Yes	Unclear	Yes
Machorowska-Pieniążek A *et al.* (2012) [53]	Yes	No	Yes	Yes	Yes	No	Yes	Yes
Oblack C *et al.* (2013) [66]	Yes	Yes	Yes	Yes	Yes	Yes	Unclear	Yes
Priya V *et al.* (2013) [54]	Yes	Yes	Yes	Yes	Yes	Yes	Unclear	Yes
Fraiz FC *et al.* (2014) [55]	Yes	No	Yes	Yes	Yes	Yes	Unclear	Yes
Shah R *et al.* (2014) [43]	Yes	Yes	Yes	Yes	Yes	Yes	Unclear	Yes
Suja J *et al.* (2014) [56]	Yes	Yes	Yes	Yes	Yes	Yes	Unclear	Yes
Bergendal B *et al.* (2015) [57]	Yes	Yes	Yes	Yes	Yes	Yes	Unclear	Yes

Knobloch LA *et al.* (2018) [59]	Yes	No	Yes	Yes	Yes	Yes	Yes	Yes
Celli D *et al.* (2018) [15]	Yes	Yes	Yes	Yes	Yes	Yes	Unclear	Yes
Kuźniarski A *et al.* (2018) [16]	Yes	Yes	Yes	Yes	Yes	Yes	Yes	Yes
Yajing L *et al.* (2019) [61]	Yes	No	Yes	Yes	Yes	Yes	Yes	Yes
Ierardo G *et al.* (2019) [62]	Yes	Yes	Yes	Yes	Yes	Yes	No	Yes
Szemraj-Folmer A *et al.* (2019) [63]	Yes	No	Yes	Yes	Yes	Yes	Yes	Yes
Wimalarathna A. A. A. K *et al.* (2020) [14]	Yes	Yes	Yes	Yes	Yes	Yes	Unclear	Yes
Gonzaga Luiz H. *et al.* (2021) [42]	Yes	Yes	Yes	Yes	Yes	Yes	Yes	Yes

Supplementary Table 2. Responses to the Joanna Briggs Institute Critical assessment items for Case Series.

**CASE SERIES**										
Author(s)/year	Item No.									
	1	2	3	4	5	6	7	8	9	10
Fotso J *et al.* (2009) [65]	Yes	No	No	No	Yes	Yes	Yes	Yes	Yes	No
Kościelska N *et al.* (2016) [58]	Yes	Yes	Yes	No	Yes	Yes	Yes	Yes	Yes	No
Schanbl D *et al.* (2018) [60]	Yes	Yes	Yes	No	Yes	Yes	Yes	Yes	Yes	No
